# Ketamine for treatment-resistant post-traumatic stress disorder: double-blind active-controlled randomised crossover study

**DOI:** 10.1192/bjo.2025.10854

**Published:** 2025-10-01

**Authors:** Ben Beaglehole, Paul Glue, Shona Neehoff, Shabah Shadli, Neil McNaughton, Bridget Kimber, Chrissie Muirhead, Aroha de Bie, Rachel Day-Brown, Natalie J. Hughes-Medlicott

**Affiliations:** Department of Psychological Medicine, University of Otago, Christchurch, New Zealand; Department of Psychological Medicine, University of Otago, Dunedin, New Zealand; Department of Psychology, University of Otago, Dunedin, New Zealand; School of Psychology, Charles Sturt University, Bathurst, Australia; School of Pharmacy, University of Otago, Dunedin, New Zealand

**Keywords:** Ketamine, post-traumatic stress disorder, trauma and stressor-related disorders, psychopharmacology, fentanyl

## Abstract

**Background:**

Ketamine is a promising treatment for post-traumatic stress disorder (PTSD), but further research is required to extend early findings.

**Aims:**

To determine the short-term efficacy and tolerability of intramuscular (i.m.) ketamine compared with i.m. fentanyl for treatment-resistant PTSD symptoms.

**Method:**

We completed a randomised double-blind psychoactive-controlled study with single doses of i.m. racemic ketamine 0.5 mg/kg or 1.0 mg/kg or i.m. fentanyl 50 μg (psychoactive control). Eligible participants were aged between 18 and 50 years old and had treatment-refractory PTSD. The primary efficacy measure was the Impact of Events Scale – Revised (IESR), and tolerability was measured with the Clinician-Administered Dissociative States Scale. Analysis of variance with dose and time as repeated measures was used to assess the effects of drug treatment on total IESR and Clinician-Administered Dissociative States Scale scores.

**Results:**

Thirty-three participants completed the study (26 females, mean age 34.5 years). Ketamine, particularly at 1 mg/kg, was associated with substantially reduced IESR ratings, with some effect remaining after 1 week. Ketamine was also associated with short-term dissociative and cardiovascular effects.

**Conclusions:**

We provide preliminary support for the efficacy and tolerability of i.m. ketamine in a community sample of individuals with PTSD. Further work is required to establish the optimal dosing regimen and longer-term role of ketamine in treatment of PTSD, but our findings are encouraging given the well-known of treatments in this area.

Approximately 70% of individuals worldwide are exposed to at least one traumatic incident.^1^ Rates of post-traumatic stress disorder (PTSD), which vary according to trauma type and frequency of trauma exposure, are greatest following exposure to intimate partner sexual violence and represent a high population burden.^1^ Recommended psychotherapies for PTSD include trauma-focused cognitive–behavioural therapy, prolonged exposure therapy, cognitive processing therapy and eye movement desensitisation reprocessing therapy.^2,3^ First-line medication options (selective serotonin reuptake inhibitors) have only a small positive effect compared with placebo.^4^ This has prompted research on novel treatment options such as ketamine^5^ and methylenedioxymethamphetamine (MDMA).^8,9^ MDMA is typically provided as part of an assisted psychotherapy package to facilitate trauma processing, whereas randomised controlled trials (RCT) of ketamine for PTSD have provided ketamine without therapy^6,7,10^ or as part of a psychotherapy package.^11,12^

## Ketamine for PTSD

We are aware of five previous RCTs evaluating ketamine for PTSD. The first of these, by Feder et al (*n* = 41), compared a single dose of intravenous (i.v.) ketamine with midazolam for chronic PTSD and reported that 0.5 mg/kg ketamine was associated with rapid improvements in PTSD symptoms, with benefits frequently persisting beyond 24 h.^10^ A follow-up study (*n* = 30) tested the efficacy and safety of repeated i.v. infusions with the same ketamine dose (0.5 mg/kg) over two consecutive weeks and confirmed that ketamine was well tolerated, with greater improvements in the ketamine group.^7^ In both of these studies, the ketamine arms included greater numbers of female than male participants, and the most frequent cause of trauma was sexual assault and molestation. Abdallah et al completed the largest RCT of ketamine for PTSD to date in mostly male veterans and active-duty military personal (*n* = 158).^6^ This study compared i.v. placebo (normal saline) with 0.2 mg/kg and 0.5 mg/kg ketamine; it did not report significant group-by-time interactions, although 0.5 mg/kg ketamine was found to be beneficial for depressive symptoms. Pradhan et al completed two RCTs of ketamine for PTSD (*n* = 10 and 20) in predominantly female participants comparing a single dose of 0.5 mg/kg i.v. ketamine and Trauma Interventions using Mindfulness-Based Extinction and Reconsolidation (TIMBER) psychotherapy with i.v. saline and TIMBER psychotherapy.^11,12^ Emotional, physical and sexual abuse were the predominant trauma types in these studies. The key findings were prolonged duration of positive effects in the ketamine and TIMBER treatment arms.

Further work evaluating ketamine for PTSD is desirable. Key issues to investigate include the presence and specificity of ketamine response in patients with PTSD, predictors of response, and optimal dosing and duration of treatment. In this study, we evaluated two ketamine doses compared with a fentanyl control in a community PTSD sample. We hypothesised that each ketamine dose would reduce PTSD scores more than the fentanyl control.

## Method

This study was registered with the Australian and New Zealand Clinical Trial Registry (ACTRN12619000311156). The authors assert that all procedures contributing to this work comply with the ethical standards of the relevant national and institutional committees on human experimentation and with the Helsinki Declaration of 1975, as revised in 2013. All procedures involving human participants and/or patients were approved by the Central Health and Disability Ethics Committee (19/CEN/21, approval date 17 April 2019). All participants gave written informed consent.

The CONSORT checklist detailing the location of key design features is provided in the Supplementary Material available at https://doi.org/10.1192/bjo.2025.10854. This research is part of a wider study that recruited patients with treatment-resistant depression (TRD), PTSD, obsessive–compulsive disorder and spider phobia in separate cohorts to evaluate the effects of ketamine on electroencephalogram (EEG) biomarkers linked to anxiety and clinical outcomes.^13^ Only the PTSD cohort and clinical data are reported in this paper. This was a randomised double-blind psychoactive-controlled study in patients with treatment-refractory DSM-5 PTSD (inadequate response to at least two adequate trials of antidepressants and one trial of relevant psychotherapy). Treatment and data collection occurred in two locations (Dunedin and Christchurch, New Zealand). Patients were screened by a psychiatrist (B.B. or P.G.) and interviewed using a structured clinical interview, the Mini-International Neuropsychiatric Interview.^14^

Patient inclusion criteria included having an Impact of Events Scale – Revised (IESR^15^) score of ≥33 (consistent with a high likelihood of PTSD diagnosis when the rating scale score is used for diagnostic purposes),^16^ having treatment-refractory PTSD (as defined above), being aged between 18 and 50 years (to minimise age-related variation in the EEG component of the study) and having good overall health. The study cohort was stratified into those with PTSD but no major depressive disorder (MDD; Montgomery–Åsberg Depression Rating Scale (MADRS^17^) score of ≤20 at screening) and those with comorbid PTSD and TRD (failure to respond to at least two antidepressants and psychotherapy and MADRS score of ≥20 at screening) for subanalysis. Exclusion criteria included evidence of severe or chronic medical disorders, past or current diagnoses of schizophrenia, bipolar disorder or current psychotic symptoms, and current significant suicidal ideation; patients were also excluded if they were pregnant or lactating, had substance use disorder or dependence in the past 6 months, or had a prior history of seizures or head injury. The exclusion criteria were intended to ensure that potential participants were stable and able to tolerate study participation in a psychiatric setting. Ethnicity was ascertained by self-report and from health records. Patients provided signed informed consent before screening and were assessed as suitable to participate on the basis of a review of medical history, safety laboratory tests (complete blood count, electrolytes, pregnancy test for patients who were capable of becoming pregnant), negative urine drug screening and vital signs. Patients were asked to provide a referral from a general practitioner or psychiatrist who knew them well and could confirm the medical diagnosis and prior treatment. Patients were permitted to remain on current medication regimens and to continue with ongoing psychotherapy; however, no new treatments were to be started or changed during the study.

Study treatments included single doses of racemic ketamine 0.5 mg/kg, 1.0 mg/kg or fentanyl 50 μg (psychoactive control). Fentanyl was chosen as the control because some of its acute side-effects (euphoria and sedation) overlap with effects of ketamine. The ketamine doses were selected after previous work investigating the ketamine dose–response relationship in other anxiety disorders.^18,19^ The study treatments were administered as intramuscular (i.m.) injections in the deltoid muscle by the study psychiatrists (B.B. and P.G.). The i.m. route was chosen as this is less invasive than i.v. administration, and response to ketamine was found to be comparable between these routes in a pilot study.^20^ Study drugs were given according to a computer-generated random code with balanced randomisation (*N* = 4–6 across the six possible drug orders), using a three-way within-participant double-blind active-controlled crossover design. There were three dosing sessions, each separated by at least 1 week. Dosing was delayed if the participants’ IESR was reduced by <50% compared with their prior pre-dose score or because of other factors such as illness, COVID-19 lockdowns or scheduling challenges. A 10-min relaxation EEG test was obtained pre-dose and 2 h and 24 h after each dosing session to assess the timing of EEG changes in response to study treatments (data to be presented elsewhere). Patients were monitored in the research clinic for a minimum of 2 h post-dose, with vital signs obtained pre-dose and at 15, 30, 45, 60, 90 and 120 min post-dose. PTSD symptoms severity was evaluated using the IESR pre-dose and at 1, 2, 24 and 168 h (∼7 days) post-dose. The original study protocol specified the Clinician-Administered PTSD Scale-5 at 24 h (CAPS-5)^21^ as the primary outcome. Before the study commenced, we switched to the self-report Impact of Events Scale – Revised (IESR)^15^ at 24 h, because this takes approximately 4 min, whereas the CAPS-5 takes 45–60 min (the outcome measure is repeated three times over the dosing period). The IESR is a 22-item scale; each item is rated on a five-point scale (0–4), with higher scores associated with greater subjective distress. Questions are aligned with DSM-IV PTSD symptom criteria,^22^ and scores for the intrusion, avoidance and hyperarousal subscales can be calculated. The IESR anchors symptoms to the past 7-day period. When the IESR was repeated at shorter time intervals for outcome measurement purposes, participants were advised to anchor symptom reporting to the period since the IESR had last been repeated. The IESR includes sleep items; as there had been no new sleep opportunity at the 1 and 2-h post-dose time points, scores for these items were likely to be rated low. The participants were blind to treatment allocation. Other assessments were completed by nurses blind to treatment allocation. Responder rates (patients with reductions in IESR scores ≥50%) were evaluated at 24 h post-dose. Tolerability assessments included reported adverse events throughout the study and Clinician-Administered Dissociative States Scale (CADSS^23^) scores pre-dose and 30 and 60 min post-dose. Bladder symptoms were monitored using the Bladder Pain/Interstitial Cystitis Symptom Score.^15^ Owing to our previous experience in treating patients with ketamine, we implemented a protocol of administering 4 mg of oral ondansetron 1 h before dosing to reduce the incidence of nausea and vomiting. Maintenance of blinding in participants and raters was not assessed. Following the randomised part of the study, participants were eligible to participate in a 6-week course of oral ketamine. Findings from this second part of the study will be reported later.

We assessed cognition using orientation questions and trail-making tests before dosing, because changes in cognition (memory impairment and executive functioning) have been reported when ketamine is used recreationally at high doses.^24^ Before discharge from the research clinic, 2 h after dosing, we assessed participants’ level of orientation, blood pressure and heart rate to check these were ≤120% of baseline, as well as checking that participants were able to walk unassisted, were feeling physically well, and were not significantly sedated or distressed. Blinded safety data were reviewed during the study by an independent data safety monitoring board.

Summary statistics were calculated and are reported for demographic, vital signs and rating scale data. Categorical variables are reported using counts and percentages. The full IESR scale was the primary outcome measure, but we also report the effects of study treatments on the three IESR subscales (intrusiveness, avoidance and hyperarousal. We used data from Feder et al 2014^10^ to calculate sample size (with 33 participants, mean IESR difference for ketamine versus placebo in the first dosing period = 8.6, s.d. = 17, alpha = 0.05, statistical power: 81%).

Analysis of variance (ANOVA) with dose and time as repeated measures was used to assess the effects of drug treatment on total IESR scores and CADSS scores. The data were first checked for skew and transformed to remove any relationship between means and standard errors. In the case of IESR scales, we calculated IESR’ = (IESR + 0.5)^0.8^. The 0.5 value was added as the data contained zero values.^25^ In the case of CADSS, which showed range compression for small values, we applied the angular transform, first converting the CADSS score to a proportion of its range and then outputting the transform values in degrees rather than radians. That is, we calculated CADSS’ = asin(sqrt(CADSS/108)) × 180/pi(). This results in numbers on a similar scale, with a CADSS of 40 being converted to a CADSS’ of 37.5°. The ANOVA was run using IBM SPSS Statistics version 28.0.1.0 for Windows 10, which automatically extracted orthogonal polynomial components of the dose and time factors. The resultant components (including interaction components) had one numerator degree of freedom and so were not affected by sphericity or related repeated-measures problems, with each component tested against its own error term. Orthogonal polynomials are independent of each other, purely descriptive and do not assume any particular underlying form of mathematical function. The use (and graphing) of transforms and orthogonal polynomials is described further in ref. ^26^

## Results

**Fig. 1:**
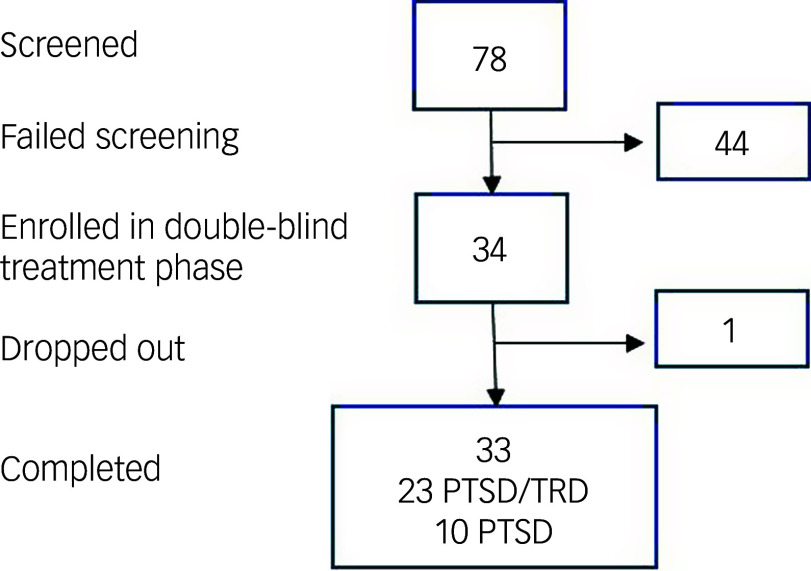
Consort diagram of participant flow in this study. PTSD, post-traumatic stress disorder; PTSD/TRD, post-traumatic stress disorder with comorbid treatment-resistant depression.

We completed the study after recruiting 34 participants from a screening cohort of 78 patients (Fig. 1) between 22 May 2021 and 8 February 2024. The main reasons for failing screening were psychiatric and physical comorbidities meaning that the exclusion criteria were met. During the study, one individual who was unable to tolerate acute medication side-effects dropped out, so the final analysed data-set comprised 33 patients. Demographic and clinical characteristics of the population are listed in Table 1.

**Table 1:** Demographic and clinical details of participants who completed the study

	All patients (*n* = 33)	Non-comorbid PTSD (*n* = 10)	Comorbid PTSD and TRD (*n* = 23)
Age in years, mean (s.d.)	34.5 (9.7)	34.2 (8.4)	32.1 (10.6)
Weight in kg, mean (s.d.)	81.2 (21.3)	80.0 (18.7)	81.8 (24.4)
Gender	26 female, 7 male	10 female, 0 male	16 female, 7 male
Ethnicity	31 New Zealand European, 2 Māori	9 New Zealand European, 1 Māori	22 New Zealand European, 1 Māori
Baseline IESR score (s.d.)	53.8 (11.2)	58.0 (7.7)	50.1 (16.1)
Baseline MADRS score (s.d.)	17.5 (5.5)	13.3 (3.9)	22.6 (4.4)
Number of failed antidepressants, mean (s.d.)	3.3 (1.6)	2.7 (0.8)	3.6 (1.8)

IESR, Impact of Events Scale – Revised; MADRS, Montgomery–Åsberg Depression Rating Scale; PTSD, post-traumatic stress disorder; TRD, treatment-resistant depression.

The participants who completed the study comprised 26 females and seven males. Their mean age was 34.5 years (range 19–50 years; Table 1), and 31 of the 33 participants were New Zealand European. The mean baseline IESR scores between patients with and without comorbid TRD (58.0 *v*. 50.1) were not statistically significantly different (unpaired *t*(31) = 1.47, *P* = 0.15). The majority of participants in both samples were affected by childhood sexual trauma, although trauma histories included other childhood trauma and traumas occurring in adulthood. There was significant psychiatric comorbidity, with 23 patients having current TRD and six having historical MDD, as well as eight with generalised anxiety disorder, seven with social anxiety disorder, four with agoraphobia and one with obsessive–compulsive Disorder. The mean number of failed antidepressants before to enrolment was 3.3 (s.d. 1.5) (Table 1), but the majority of participants continued on antidepressants (singly and in combination) during the study.

Sixty-four per cent of dosing sessions occurred 7 days post-dose, 18% with a 2-week gap, 14% with a 3-week gap, and 5% with a gap of 4 weeks or longer. Reasons for treatment postponement included the Covid lockdown, scheduling challenges, or participants’ IESR scores being <50% reduced compared with their prior pre-dose score. An analysis for order effects found significant effects on general level of IESR across a session (i.e. a pre-dose effect) but no variation in the size of the medication effect within a session. We also checked baseline IESR scores before and after dosing and found that the pre-dose IESR score for the subsequent dosing period was 2.3% greater than the baseline score (s.d. = 27.2) following fentanyl, 21.9% less than the baseline score (s.d. = 31.6) following 0.5 mg/kg ketamine, and 16.4% greater than the baseline score (s.d. = 32.0) following 1 mg/kg ketamine.

**Fig. 2:**
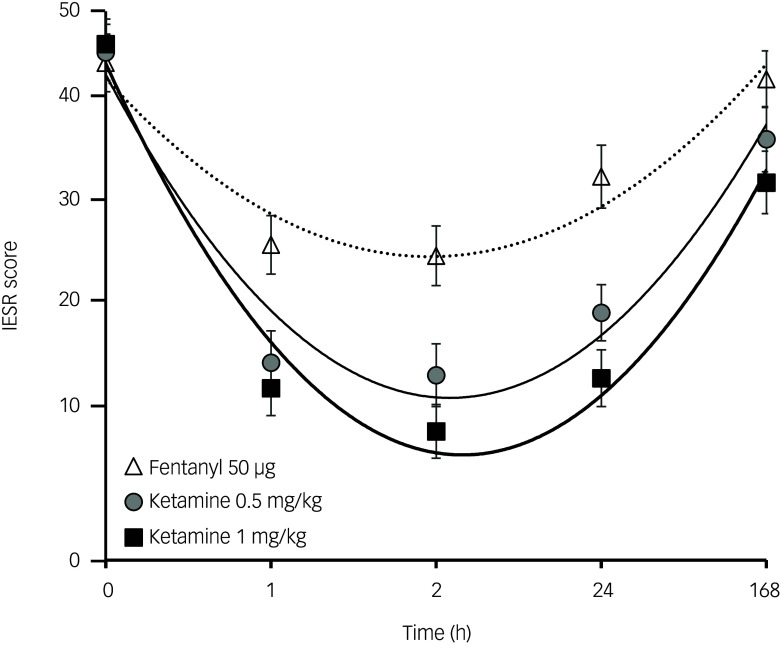
Mean (s.e.m.) Impact of Events Scale – Revised (IESR) response to study treatments. The fitted curves show the significant linear plus quadratic trend components detected in the analysis of variance. The non-linear IESR axis is a result of the transform used to normalise error variance. The 0 h time point was immediately before dosing.

Mean IESR scores over time by study treatment for all participants are shown in Fig. 2, and mean IESR subscale profiles are shown in Supplementary Fig. 1. The reduction in IESR scores seemed to be dose-related compared with the fentanyl treatment arm (dose[linear) *F*(1,30) = 26.61, *P* < 0.0001; dose[linear] × time[linear] *F* (1,30) = 12.59, *P* = 0.001; dose[linear] × time[quadratic] *F*(1,30) = 31.95, *P* < 0.001). The apparent non-linearity of the dose effect was not significant (dose[quadratic) *F*(1,30) = 1.94, *P* = 0.174; dose[quadratic] × time[linear] *F*(1,30) = 0.098; dose[quadratic] × time[quadratic] *F*(1,30) = 0.30). However, although the magnitude of response seemed to be greater and more sustained in duration after ketamine at 1 mg/kg compared with 0.5 mg/kg, the difference between these two ketamine doses was not statistically significantly different in a *post hoc* ANOVA (dose[deviation] *F*(1,30) = 3.84, *P* = 0.06; dose[deviation] × time[linear] *F*(1,30) = 2.87, *P* = 0.10; dose[deviation] × time[quadratic] *F*(1,30) = 1.06). The proportions of treatment responders (>50% reduction at 24 h compared with baseline) were 18% after fentanyl, 64% after ketamine 0.5 mg/kg and 81% after ketamine 1.0 mg/kg. All three IESR subscales showed similar time and dose–response profiles to those of the combined scale (Supplementary Fig. 1). Mean IESR responses were similar in patients with and without comorbid TRD (Supplementary Fig. 2).

For 11 patients, we had parallel pre- and 2 h-post-dose IESR and MADRS data. IESR and MADRS shared about 40% of their variance (*r* = 0.645). Despite the small *N*, ketamine significantly affected IESR (dose[linear] *F*(1,10) = 6.018, *P* = 0.034; dose[linear] × time *F*(1,10) = 29.688, *P* < 0.001) and, to a lesser extent, MADRS (dose[linear] *F*(1,10) = 2.516, non-significant; dose[linear] × time *F*(1,10) = 9.190, *P* = 0.013). IESR covaried on MADRS retained some significance (dose[linear] *F*(1,10) = 3.430, *P* = 0.094, non-significant; dose[linear] × time *F*(1,10) = 6.845, *P* = 0.026). This is consistent with about half of ketamine’s effect on IESR being due to the same underlying factor as the change in MADRS (direction of causalities unknown) but about another half of ketamine’s effect on IESR being significant and unrelated to MADRS.

### Safety and tolerability

**Fig. 3:**
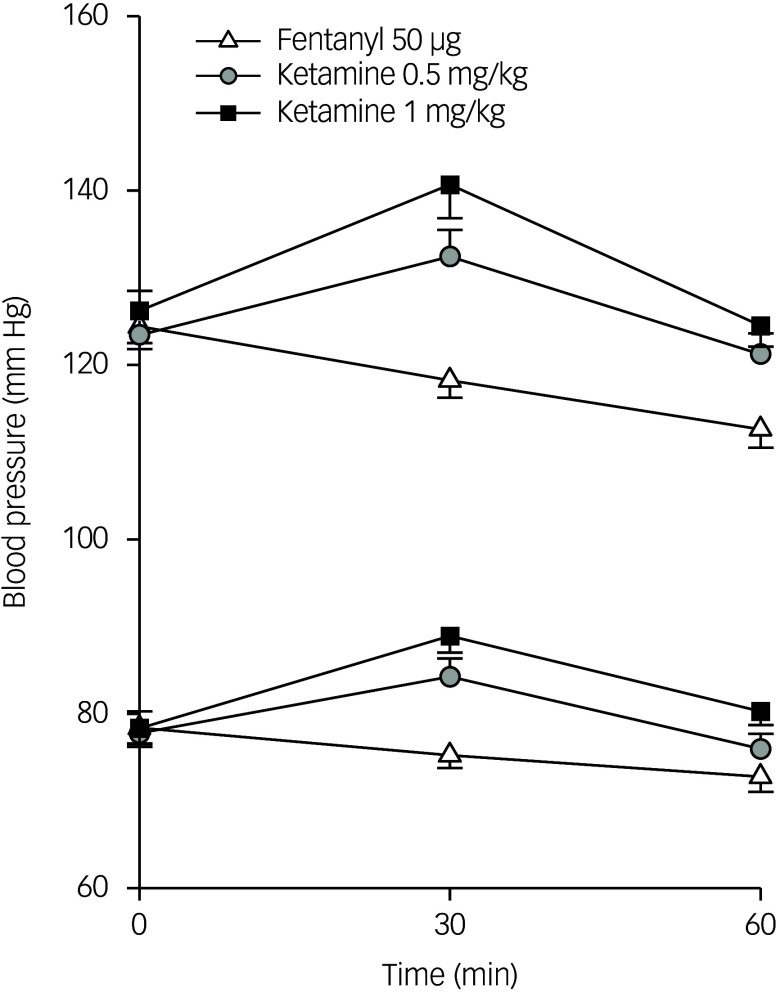
Mean (s.e.m.) blood pressure response to study treatments.

Blood pressure changes after dosing are shown in Fig. 3. There were negligible changes after fentanyl. Thirty minutes after ketamine dosing, mean changes in systolic blood pressure were −6.2, 9.0 and 14.5 mmHg for the fentanyl, ketamine 0.5 mg/kg and ketamine 1.0 mg/kg groups, respectively; the corresponding changes in diastolic blood pressure were −3.2, 6.5 and 10.6 mmHg, respectively (Fig. 3). Blood pressure values trended downwards by 60 min. All patients reported dissociative symptoms after ketamine dosing, starting approximately 3–5 min after each i.m. injection, with the intensity reaching a peak at around 15–30 min and then slowly decreasing.

CADSS scores did not increase after fentanyl, were increased after 0.5 mg/kg ketamine, and were highest after the 1.0 mg/kg dose of ketamine, (Supplementary Fig. 3) with a peak 30 min post-dose. Both the residual increase in CADSS at 60 min and the peak effect at 30 min had clear linear components of dose (dose[linear] × time[linear] *F*(1,32) = 25.49, *P* < 0.001; dose[linear] × time[quadratic] *F*(1,32) = 25.49, *P* < 0.001), but with the peak effect of 0.5 mg/kg of ketamine being closer to that of 1.0 mg/kg ketamine than to that of fentanyl (dose[quadratic] × time[quadratic] *F*(1,32) = 7.76, *P* = 0.009).

### Adverse events

The most common reported adverse event after dosing with ketamine was dissociation, usually starting within 3–5 min of ketamine being administered. Many patients reported having blurred vision, that they experienced a feeling of being light-headed or sedated, and that their lips felt numb. Owing to pre-dosing with ondansetron, it is likely that rates of nausea and vomiting were reduced. The most commonly experienced adverse event after fentanyl was a mild level of drowsiness that was short lived. For the Bladder Pain/Interstitial Cystitis Symptom Score, Trail Making Test A and Trail Making Test B data, there were no significant effects of drug (all *F*(2,29) < 0.78, *P* > 0.38) or diagnosis (all *F*(2,29) < 2.1, *P* > 0.14), nor were there any interactions of the two (all *F*(2,29) < 1.03, *P* > 0.35). No patient died or experienced a serious adverse event.

## Discussion

New and effective treatments for PTSD are needed because of inadequate responses to existing treatments and the high burden of illness. We report that i.m. ketamine is more efficacious than i.m. fentanyl for PTSD. Ketamine was associated with dose-dependent dissociative symptoms and cardiovascular changes but was otherwise well tolerated without serious adverse effects.

Several of our findings are noteworthy and deserve consideration alongside those of previous studies. Feder et al reported benefits from 0.5 mg/kg ketamine infusions in predominantly female PTSD patients.^7,10^ Similarly, Pradhan et al reported the benefit of adding 0.5 mg/kg i.v. ketamine infusions to TIMBER psychotherapy.^11,12^ By contrast, Abdallah et al did not report benefits from 0.2 mg/kg and 0.5 mg/kg i.v. ketamine infusions in a predominantly male sample of veterans and active-duty personnel.^6^ Taken together, these results raise questions about the durability and specificity of ketamine for PTSD, as well as whether gender and trauma type influence response, and the optimal ketamine dosing for PTSD.

We compared 0.5 mg/kg and 1 mg/kg i.m. ketamine doses with psychoactive control drug fentanyl. Ketamine, particularly at 1 mg/kg, was associated with substantial reductions in IESR ratings at 24 h, with some effect remaining after 1 week. There were also more responders to the 1 mg/kg ketamine dose than to the 0.5 mg/kg dose. Taken together, these findings suggest the possible presence of a dose-effectiveness gradient, and that doses higher than 0.5 mg ketamine may be more effective for PTSD symptoms, although the optimal trade-off between side-effects and dose to maximise efficacy is still not clear. A previous study compared i.v., i.m. and subcutaneous routes for ketamine in depression and reported that all three routes resulted in comparable antidepressant effects, although peak plasma concentrations after i.v. dosing were approximately double those recorded after i.m. and subcutaneous dosing.^20^ Therefore, efficacy may not relate to peak plasma ketamine concentrations, and other methods of delivery including oral dosing may also be beneficial for patients with PTSD, with a lower liability for dissociative side-effects. Abdallah et al^6^ completed the largest ketamine PTSD study to date (*n* = 158). We speculate that the lack of efficacy in this study may have been related to too-low ketamine dosing for this patient group. Their sample of predominantly male military veterans and active-duty personnel differed from civilian samples and may have contributed to non-responsiveness. Prazosin treatment for PTSD in veterans requires higher doses than those used for civilians,^27,28^ and this might also be the case for ketamine treatment. In support of this premise, the ketamine and TRD literature suggests that variable and higher ketamine doses are more effective than fixed and lower ketamine doses.^29,30^

Our study and those of Feder et al^7,10^ and Pradhan et al^11,12^ reported benefits from ketamine treatment for patients with PTSD. These studies evaluated civilian samples with greater proportions of females. In addition, the most common traumas in these studies were sexual, emotional and/or physical abuse.^7,10–12^ Our study was not powered to distinguish effects according to gender or trauma type, but this topic will be of interest if continued differences in responses according to study population are reported. In addition, scores on all subscales of the IESR improved with ketamine, but larger studies are required to confirm the symptom profile of response to ketamine treatment. Rytwinski reported that 52% of patients in 57 studies who had PTSD had co-occurring MDD.^31^ We report that positive responses to ketamine were present in both the PTSD-only group and the PTSD with comorbid TRD group. Psychiatric comorbidity is common, and evidence of effectiveness across diagnostic boundaries is therefore reassuring. The EEG findings from this study (to be reported later) may help to determine whether there are EEG predictors of response and whether these are diagnosis-specific^32^ or cross diagnostic boundaries.

The first-line treatment for PTSD is trauma-focused psychotherapy.^2,3^ Conceptually, concurrent ketamine treatment may provide additional therapeutic benefits and facilitate the trauma-exposure work. Although the literature supporting combinations of ketamine and psychotherapy for psychiatric disorders is promising, the studies reported have frequently lacked design features to enable them to determine whether the addition of psychotherapy improves outcomes compared with ketamine treatment alone.^33^ Consequently, we recommend larger studies evaluating combinations of ketamine and manualised psychotherapies to better understand the role of psychotherapy alongside ketamine treatment for PTSD.

In this paper, we report findings from the initial randomised phase of our study. PTSD response was tracked using the self-rated IESR as opposed to a clinician-rated scale, although initial diagnosis was confirmed using the Mini-International Neuropsychiatric Interview. The CAPS-5^21^ is the gold-standard clinician-rated instrument for assessment of PTSD, but it is not suitable for repeated assessments in close succession as it takes 45–60 min to complete. Sleep quality was assessed using the IESR and other PTSD measures including the commonly used alternative (the PTSD Checklist for DSM-5).^34^ This meant that the IESR scores at 1 and 2 h were likely to have underestimated PTSD severity, given the lack of sleep opportunity since the last rating.

Although we found that PTSD responded to ketamine, the chronic nature of PTSD suggests that longer-term interventions are required. This may entail the addition of psychotherapy^33^ or longer courses of ketamine. Data from the oral continuation phase of our study will provide knowledge in this area and will be reported separately later. Many of our participants were female with childhood sexual trauma. Given the high burden of PTSD associated with intimate-partner sexual trauma,^1^ specific treatments for PTSD caused by sexual trauma are desirable. However, the lack of diversity in gender and trauma type in our study limits the degree to which our findings can be generalised to other settings.

We did not assess blinding of participants and raters, but issues with expectation bias and failure of blinding in ketamine and psychedelic trials are well recognised.^35^ The crossover design of this study accentuated the difficulty of maintaining blinding, because each participant received all treatments over the study period and could make comparisons between treatments. The crossover design also carried the risk of carryover effects if benefits persisted into the following dosing period. These were present for the two ketamine doses, although the magnitude of IESR change was not dependent on treatment order.

We chose fentanyl as the control in this study because some of its effects (euphoria and sedation) overlap with side-effects of ketamine. More frequently, saline and midazolam are chosen as controls in ketamine studies. Saline is clearly limited owing to the absence of any dissociation. Midazolam is complicated because it may provide therapeutic effects and does not cause significant dissociation.^36^ On the basis of the differences in CADSS scores, we suspect that fentanyl at 50 μg did not provide an adequate blind and would not recommend its use for this purpose in future studies (although we do not have a alternative without limitations to recommend). Nonetheless, the treatment-resistant nature of the participants’ conditions provides some reassurance with respect to the value of the ketamine-related changes we report. In addition, EEG work to be published later may provide a biological explanation for ketamine responsiveness beyond that achieved through any expectation bias. We did not assess side-effects with a structured questionnaire. Consequently, the side-effects reported may have been underestimated compared with those that would have been identified in a more formal evaluation.

Our study provides support for the preliminary effectiveness of ketamine for the treatment of PTSD in a civilian population, as well as suggesting the possibility of a dose-effectiveness gradient, although more work is required to clarify the optimal dosing regimen for PTSD. In addition, longer-term studies will be required to clarify the ongoing role of ketamine treatment in PTSD, but our findings are encouraging given well-known limitations of treatments in this area.

## Supporting information

Beaglehole et al. supplementary material 1 Beaglehole et al. supplementary material

Beaglehole et al. supplementary material 2 Beaglehole et al. supplementary material

Beaglehole et al. supplementary material 3 Beaglehole et al. supplementary material

Beaglehole et al. supplementary material 4 Beaglehole et al. supplementary material

## Data Availability

Requests for the analytic code and data that support this study will be considered by the corresponding author (B.B.), and the materials will be made available upon reasonable request.

## Supplementary material

The supplementary material is available online at https://doi.org/10.1192/bjo.2025.10854
