# Supplementary material for: Ketamine for treatment-resistant post-traumatic stress disorder: double-blind active-controlled randomised crossover study
Source: BJPsych Open. 2025 Oct 1;11(6):e230. doi: 10.1192/bjo.2025.10854 (PMC12529321; doi:10.1192/bjo.2025.10854)
Supplement: Beaglehole et al. supplementary material 1 — Beaglehole et al. supplementary material [file S2056472425108545sup001.pptx]

## Slide 1
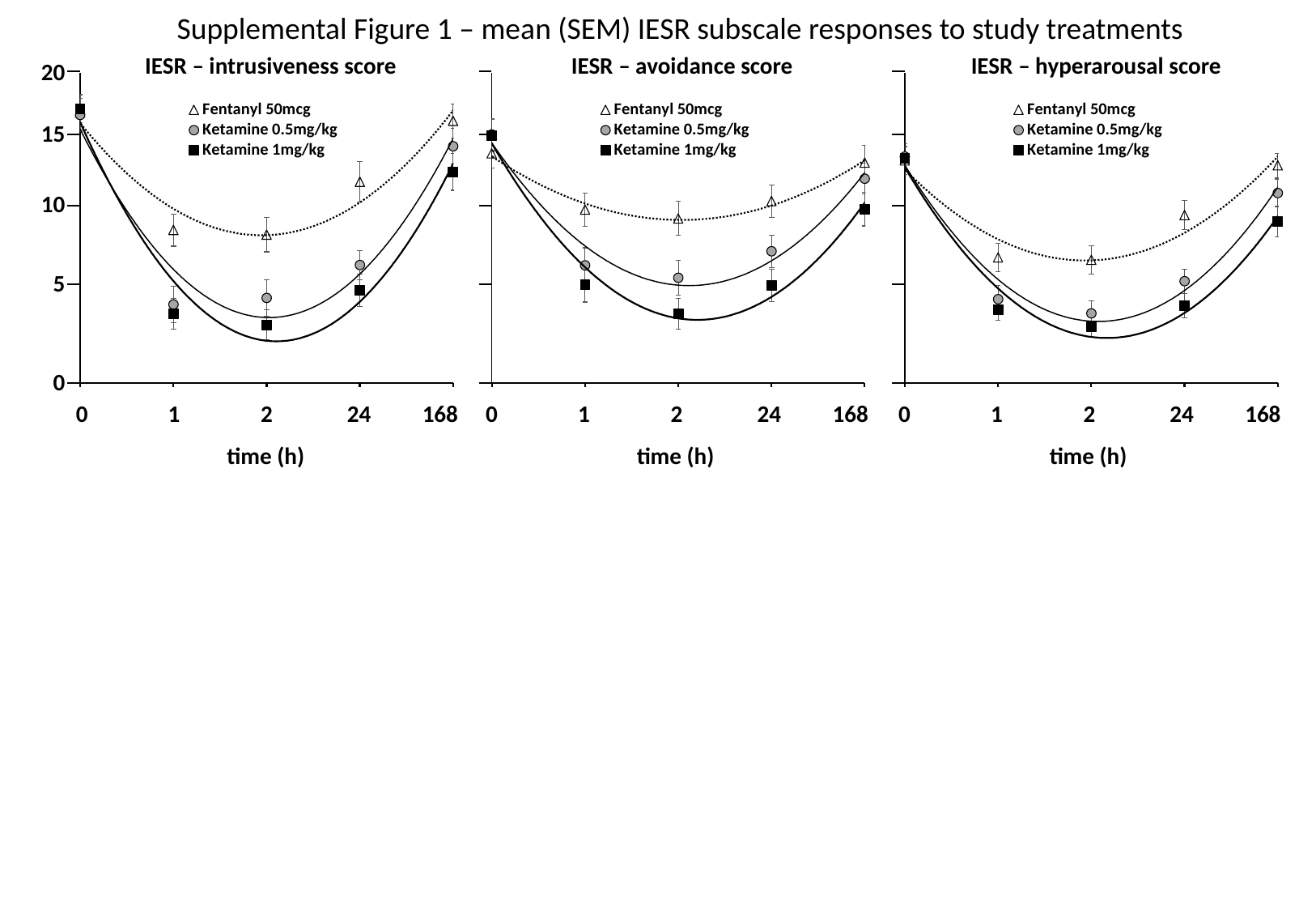

Supplemental Figure 1 – mean (SEM) IESR subscale responses to study treatments
20
15
10
5
0
IESR – intrusiveness score
IESR – avoidance score
IESR – hyperarousal score
0
1
2
24
168
time (h)
0
1
2
24
168
time (h)
0
1
2
24
168
time (h)
