# Supplementary material for: Ketamine for treatment-resistant post-traumatic stress disorder: double-blind active-controlled randomised crossover study
Source: BJPsych Open. 2025 Oct 1;11(6):e230. doi: 10.1192/bjo.2025.10854 (PMC12529321; doi:10.1192/bjo.2025.10854)
Supplement: Beaglehole et al. supplementary material 2 — Beaglehole et al. supplementary material [file S2056472425108545sup002.pptx]

## Slide 1
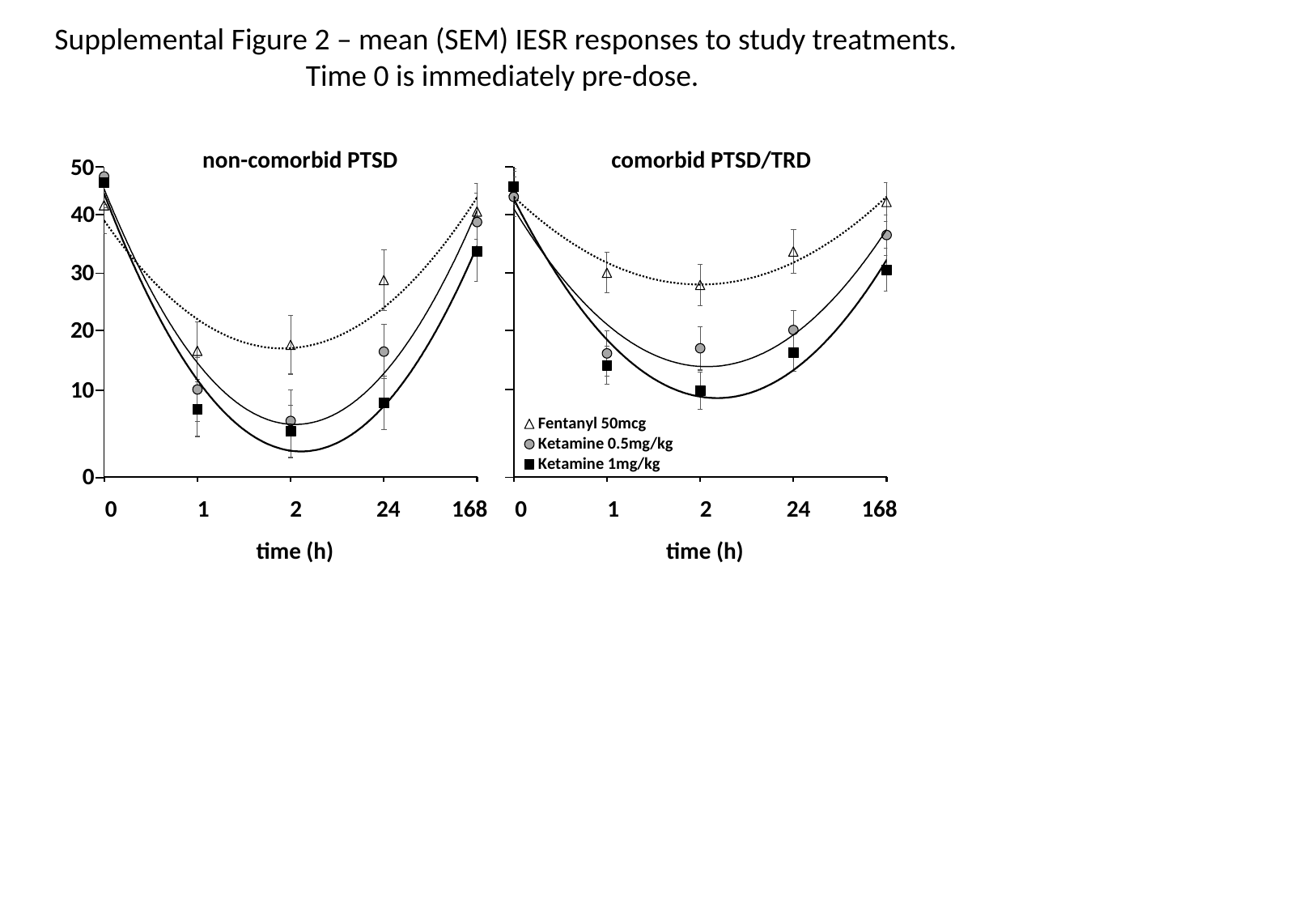

Supplemental Figure 2 – mean (SEM) IESR responses to study treatments. Time 0 is immediately pre-dose.
non-comorbid PTSD
comorbid PTSD/TRD
0
1
2
24
168
0
1
2
24
168
time (h)
time (h)
50
40
30
20
10
0
