# Supplementary material for: Ketamine for treatment-resistant post-traumatic stress disorder: double-blind active-controlled randomised crossover study
Source: BJPsych Open. 2025 Oct 1;11(6):e230. doi: 10.1192/bjo.2025.10854 (PMC12529321; doi:10.1192/bjo.2025.10854)

Supplementary Figure 3 – Mean (SEM) CADSS response to study treatments. The fitted curves show the significant linear plus quadratic trend components detected in the ANOVA. The non-linear CADSS axis is the result of the transform used to normalise error variance.

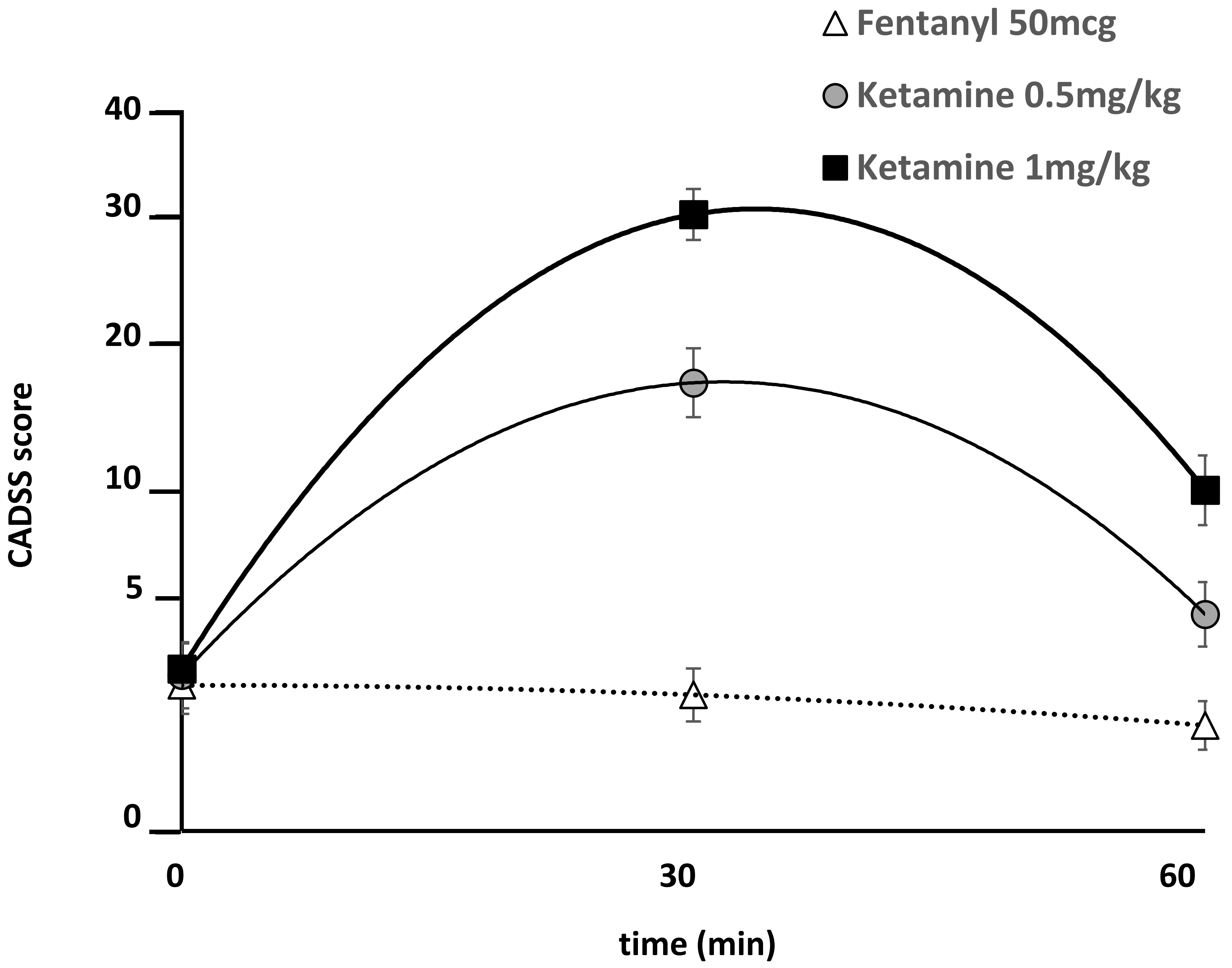

Supplement: Beaglehole et al. supplementary material 3 — Beaglehole et al. supplementary material [file S2056472425108545sup003.pdf]
